# Creating culturally-informed protocols for a stunting intervention using a situated values-based approach (*WeValue InSitu*): a double case study in Indonesia and Senegal

**DOI:** 10.1186/s12889-024-18485-y

**Published:** 2024-04-09

**Authors:** Annabel J. Chapman, Chike C. Ebido, Rahel Neh Tening, Yanyan Huang, Ndèye Marème Sougou, Risatianti Kolopaking, Amadou H. Diallo, Rita Anggorowati, Fatou B. Dial, Jessica Massonnié, Mahsa Firoozmand, Cheikh El Hadji Abdoulaye Niang, Marie K. Harder

**Affiliations:** 1 Values & Sustainability Research Group, School of Architecture, Technology and Engineering, University of Brighton, Brighton, UK; 2 Department of Environmental Science and Engineering, Fudan University, Shanghai, People’s Republic of China; 3 Department of Zoology and Environmental Biology, University of Nigeria, Nsukka, Nigeria; 4 Preventive Medicine and Public Health, Université Cheikh Anta Diop (UCAD), Dakar, Senegal; 5 Faculty of Psychology, Universitas Islam Negeri Syarif Hidayatullah, Jakarta, Indonesia; 6 Southeast Asian Ministers of Education Organization Regional Centre for Food and Nutrition (SEAMEO RECFON) Universitas Indonesia, Jakarta, Indonesia; 7 International Research Laboratory (IRL 3189) Environnement santé et sociétés/CNRS/UCAD, Dakar, Senegal; 8 Department of Medical Records and Health Information, Faculty of Health and Technology, Universitas Bandung, Bandung, Indonesia; 9 Laboratory of Cultural Anthropology, IFAN, Université Cheikh Anta Diop (UCAD), Dakar, Senegal; 10 School of Education, Languages and Linguistics, Faculty of Humanities and Social Sciences, University of Portsmouth, Portsmouth, UK; 11 Department of Learning and Leadership, IOE, UCL’s Faculty of Education and Society, University College London, London, UK

**Keywords:** Health intervention, Stunting, Shared values, Culturally-informed protocol, Cultural factors, *WeValue InSitu*

## Abstract

International development work involves external partners bringing expertise, resources, and management for local interventions in LMICs, but there is often a gap in understandings of relevant local shared values. There is a widespread need to better design interventions which accommodate relevant elements of local culture, as emphasised by recent discussions in global health research regarding neo-colonialism. One recent innovation is the concept of producing ‘cultural protocols’ to precede and guide community engagement or intervention design, but without suggestions for generating them. This study explores and demonstrates the potential of an approach taken from another field, named *WeValue InSitu*, to generate local culturally-informed protocols. *WeValue InSitu* engages stakeholder groups in meaning-making processes which ‘crystallize’ their envelope of local shared values, making them communicable to outsiders.

Our research context is understanding and reducing child stunting, including developing interventions, carried out at the Senegal and Indonesia sites of the UKRI GCRF Action Against Stunting Hub. Each national research team involves eight health disciplines from micro-nutrition to epigenetics, and extensive collection of samples and questionnaires. Local culturally-informed protocols would be generally valuable to pre-inform engagement and intervention designs. Here we explore generating them by immediately following the group *WeValue InSitu* crystallization process with specialised focus group discussions exploring: what local life practices potentially have significant influence on the environments affecting child stunting, and which cultural elements do they highlight as relevant. The discussions will be framed by the shared values, and reveal linkages to them. In this study, stakeholder groups like fathers, mothers, teachers, market traders, administrators, farmers and health workers were recruited, totalling 83 participants across 20 groups. Themes found relevant for a culturally-informed protocol for locally-acceptable food interventions included: specific gender roles; social hierarchies; health service access challenges; traditional beliefs around malnutrition; and attitudes to accepting outside help. The concept of a grounded culturally-informed protocol, and the use of *WeValue InSitu* to generate it, has thus been demonstrated here. Future work to scope out the advantages and limitations compared to deductive culture studies, and to using other formative research methods would now be useful.

## Background

Although progress has been made towards the SDG of ‘Zero Hunger by 2025’, the global rates of malnutrition and stunting are still high [1]. Over the past 20 years, researchers have implemented interventions to reduce undernutrition, specifically focussing on the first 1000 days of life, from conception to 24 months [2]. However, due to both differing determinants between countries [3, 4] as well as varying contextual factors, it is clear that no single fixed approach or combination of approaches can be relied on when implementing stunting interventions [5–7]. Furthermore, when external researchers design interventions for local areas in Low- and Middle-Income Countries (LMICs) they can often overlook relevant local cultural factors that consequently act as barriers to intervention uptake and reduce their effectiveness, such as geographical factors and the levels of migration in certain populations [8, 9], or social norms or perceptions relating to accepting outside help, and power dynamics related to gender [10–12]. The inclusion of cultural level factors in behaviour change interventions has been proposed as a requirement for effective interventions [13]. However, despite the breadth of literature highlighting the negative impacts from failing to do this, the lack of integration or even regard of local culture remains a persistent problem in Global Health Research [14], possibly hindering progress towards the SDGs. Thus, there is a need for approaches to integrate local cultural elements into intervention design.

This lack of understanding of relevant local culture, social norms and shared values also has ethical implications. The field of Global Health Ethics was predominantly developed in the Global North, in High Income Countries (HICs), embedding values common in those countries such as the prominence of individual autonomy [15, 16]. Researchers from HICs carrying out research in LMICs may wrongly assume that values held in the Global North are universal [14] and disregard some local values, such as those related to family and collective decision making, which are core to many communities in LMICs. It is therefore important for outside researchers to have an understanding of relevant local values, culture and social norms before conducting research in LMICs so as not to impose values that do not align with local culture and inadvertently cause harm or offence [16, 17]. The importance of this is compounded by the colonial history that is often present in relationships between research communities in HICs and LMICs, and the fact that the majority of the funding and leading institutions are still located in the Global North [18, 19]. Thus, conscious steps must be taken to avoid neo-colonialism in Global Health Research [20]. From a health-equity perspective, it is essential to ensure that those in vulnerable communities are not hindered from involvement in interventions to improve nutrition. Encouraging uptake by such communities could be provided if salient local shared values, norms and culture were taken into account [21].

In a recent paper, Memon et al., (2021) highlight the usefulness of first creating a cultural protocol that can precede and guide subsequent stages of community engagement or intervention design to ensure that salient local values are known to external researchers coming into the community [16]. We adopt the use of the concept of a cultural protocol, referring to locally-generated guidance about key values, norms, behaviours and customs relevant to working with the local community. However, we prefer the term, ‘culturally-informed protocol’ since this relates to only cultural elements deemed salient by the researchers, and locally, rather than any comprehensive notion of culture, nor extending beyond the research context.

Memon et al. (2021), point out links between the creation of such a protocol and existing codes of practice that have already been created for some cultures such as the Te Ara Tika, a Guideline for Māori Research Ethics [22]. Currently, research and interventions in Global Health can be informed by a stage of formative research involving one-to-one interviews, focus groups or direct observations, which can sometimes be ethnographic in nature such as within Focussed Ethnographic Studies or Rapid Assessment Procedures [23–25]. Although these methods can be effective to inform intervention designs, they have disadvantages like: can take long periods to complete [26], can be resource intensive [26] and can lack cultural acceptability [27]. These limitations may account for the frequent neglect of their use generally, highlighted by Aubel and Chibanda (2022) [14]. Additionally, none of these methods work towards making explicit local values, or towards the creation of a culturally-informed protocol. In brief, the literature suggests a need to develop alternative methods of Formative Research for understanding locally relevant cultural elements, that are less time-consuming and can generate data that is more easily translatable to intervention design. In addition, these approaches must be applicable in different cultures. Additionally, the protocols produced must be actionable and practical not only for guiding interactions between research teams but also for guiding the initial stages of intervention design.

The work presented here aims to address several of these needs. It includes an exploration of the usefulness of the *WeValue InSitu* (*WVIS*) approach because that has previously been shown, in environmental management domains, to offer a way to gather in-depth values-based perspectives from a target population [28, 29] It was first created through action research, and co-designed to enable civil society organisations to better understand and measure the values-based aspects of their work [30]. The core *WeValue InSitu* process (detailed in Table 1) involves the crystallization of shared values, with a facilitator guiding a group of participants with shared experiences, through cycles of tacit meaning-making (using a stage of photo-elicitation and triggering) [31], until they can articulate more explicitly their shared values, in concise and precise statements. These statements are then linked together in a framework by the participants. In an example case in Nigeria, the results of the *WVIS* approach hinted at the creation of a culturally-informed protocol through an analysis of the shared values frameworks to find cultural themes for the creation of an indicator tool that was used to evaluate several development scenarios based on their social acceptability [29].

**Table 1:** Activity stages in the *WeValue InSitu* (*WVIS*) shared values approach

Stage		Description
Creation of localised trigger list		The trigger list is a list of values statements that act as prompts for participants. The list is created through interviewing local researchers and individuals familiar with local culture, on the priorities important to local communities. These are open-coded for emerging concepts of shared values, and then the draft list is then cross-referenced with a global reference version (which was built up over 10 years of research) to ensure no important values concepts are missing. Local researchers, and experienced WeValue researchers, are both indispensable here.
Creation of localised materials		The photos and mechanisms for participants to engage in the WeValue process are modified depending on local culture: sometimes this is trial and error. For example, a Bingo-style card can be used for participants to circle numbers of statements they agree with as they are read out, instead of reading and marking the list itself. The set of 60+ photos are adapted to show people and scenarios with familiar characteristics to the local participants. (Where such localised materials are carefully developed, and the groups chosen for the WeValue sessions have historical experiences in common, then the process is in-situ crystallisation and named *WVIS*.) In Kaffrine, Senegal, the usual photos used during photo elicitation were adapted, as in their current form they prompted discussions amongst the groups that moved away from their set context. Thus, a selection of small black and white icons was used in place of the colour photos.
WeValue main session 2–4 hours	Group Contextualisation	During this stage the boundaries of the participating group members are set for example “Mothers in Hyderabad”. This is absolutely essential since in the following stages the group thinking relates back to this context as a reference.
	Photo-elicitation	During this stage participants will describe something of personal ‘importance’ to them about group membership and are invited to select a ‘prop’ from a collection of localised photos.
	Triggered Meaning-making	During this stage participants select statements from the Localised Trigger List that resonate with them. Participants are then expertly facilitated to have discussions leading to tacit-explicit meaning-making cycles [31].
	Creation of Framework and Narrative	The written out, negotiated statements of shared values are placed on the table and organised into a framework showing their linkages. A participant then provides a narrative to explain how the statements link. In Kaffrine, Senegal, this process was further structured due to difficulty discussing the concepts amongst the groups. Thus, the facilitator directed the groups to discuss, Qualities, Aspirations and Interactions specifically, as these are the most common elements among *WVIS* frameworks.
subsequent Perspectives EXploration (PEX)		This stage is an optional bolt-on to the WeValue main session and involves asking the participants focus group interview style questions. Topics are left open for participants to choose the direction of the discussion. Topics chosen related to the Hub interventions e.g. early childhood learning environment, nutritional habits and perceptions of stunting.

Furthermore, it has been found that if a group of *WVIS* participants take part in a specialised focus group discussion (FGD), named Perspectives EXploration (PEX:FGD) immediately afterward the main workshop, then they easily and articulately express their perspectives on the topics raised for discussion - and with allusions to the shared values they had crystallised just prior. In an example from Shanghai, the PEX:FGDs focussed on eliciting perspectives on climate change, which were shown to be closely linked with the cultural themes existing within the shared values frameworks produced immediately prior [32]. In that case, the PEX:FGDs allowed the cultural themes generated during the main *WVIS* workshop to be linked more closely to the research question. Those results suggested that the *WVIS* plus PEX:FGD approach could be used to create a specialised culturally-informed protocol for improved intervention design.

In the study presented here, the *WVIS* approach was explored for the purpose of creating culturally-informed protocols to inform the planning of interventions within two localities of the UKRI GCRF Action Against Stunting Hub [33]. The work was carried out in two parts. Firstly, the *WVIS* main workshop was used to elicit cultural themes within the target communities, indicating key elements to consider to ensure ethical engagement. Secondly, the PEX focus group discussions focussed on life practices related to stunting which we explored for the purpose of tailoring the culturally-informed protocols to the specific purpose of improving the design of an example intervention. The Action Against Stunting Hub works across three sites where stunting is highly prevalent but via different determinants: East Lombok in Indonesia (estimated 36% of under-fives stunted), Kaffrine in Senegal (estimated 16% of under-fives stunted) and Hyderabad in India (estimated 48% of under-fives stunted) [34]. We propose that, the information about local shared values in a given site could be used to inform the design of several interventions, but for our specific exploration the focus here is a proposed ‘egg intervention’, in which pregnant women would be provided with an egg three times per week as supplement to their diet. This study proposes that identifying shared values within a community, alongside information about local life practices, provides critical cultural information on the potential acceptability and uptake of this intervention which can be used to generate culturally-informed protocols consisting of recommendations for improved intervention design.

### Aims

In this paper we aim to explore the use of the *WVIS* approach to create culturally-informed protocols to guide engagement and inform the design of localised egg interventions to alleviate stunting in East Lombok, Indonesia and Kaffrine, Senegal. We do this by analysing data about local shared values that are crystallized using the *WeValue InSitu* (*WVIS*) process to provide clear articulation of local values, followed by an analysis of life practices discussed during PEX:FGD to tailor the culturally-informed protocols for the specific intervention design.

## Methods

### Study setting

This research was exploratory rather than explanatory in nature. The emphasis was on demonstrating the usefulness of the *WeValue InSitu* (*WVIS*) approach to develop culturally-informed protocols of practical use in intervention design, in different cultural sites. This study was set within a broader shared-values workstream within the UKRI GCRF Action Against Stunting Hub project [33]. The Hub project, which was co-designed and co-researched by researchers from UK, Indonesia, Senegal and India, involves cohorts of 500 women and their babies in each site through pregnancy to 24 months old, using cross-disciplinary studies across gut health, nutrition, food systems, micro-nutrition, home environment, WASH, epigenetics and child development to develop a typology of stunting. Alongside these health studies are studies of the shared values of the communities, obtained via the *WVIS* approach described here, to understand the cultural contexts of that diverse health data. In this study the data from East Lombok, Indonesia and Kaffrine, Senegal were used: India’s data were not yet ready, and these two countries were deemed sufficient for this exploratory investigation.

### The *WVIS* approach

**Fig. 1:**
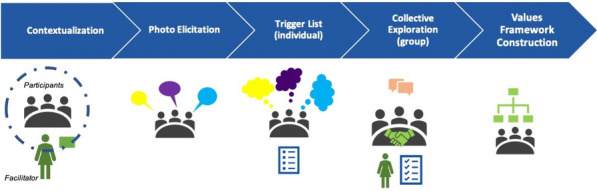
Schematic of the macro-level activities carried out during the *WeValue InSitu* (*WVIS*) main workshop session

The *WVIS* approach is a grounded scaffolding process which facilitates groups of people to make explicit their shared values in their own vocabulary and within their own frames (details in Fig. 1 and activities in Table 1). The first stage of the *WVIS* is Contextualisation, whereby the group identifies themselves and set the context of their shared experiences, for example, as ‘mothers in East Lombok, Indonesia’. Subsequently, there is a stage of Photo Elicitation, in which the group are first asked to consider what is important, meaningful or worthwhile to them about their context (e.g., ‘being mothers in East Lombok, Indonesia’) and then asked to choose photos from a localised set that they can use as props to help describe their answer to the group [29]. After this, a localised Trigger List is used. This Trigger List consists of 109 values statements that act as prompts for the group. Examples of these values statements are included below but all the statements begin with “it is important to me/us that…”. The group are asked to choose which statements within the trigger list resonate with them, and those are taken forward for group intersubjective discussion. After a topic of their shared values has been explored, the group begin to articulate and write down their own unique statements of them. These also all begin with “It is important to me/us that…”. After discussing all pressing topics, the group links the written statements on the table into a unique Framework, and one member provides a narrative to communicate it to ‘outsiders’. The *WVIS* provides a lens of each group’s local shared values, and it is through this lens that they view the topics in the focus group discussions which immediately follow, termed Perspectives EXplorations (PEX:FGDs).

This results in very grounded perspectives being offered, of a different nature to those obtained in questionnaires or using external frameworks [31]. The specific PEX:FGD topics are chosen as pertinent to stunting contextual issues, including eating habits, food systems and environments, early educational environments, and perceptions of stunting. The local researchers ensured that all topics were handled sensitively, with none that could cause distress to the participants. The data for this study were collected over 2 weeks within December 2019–January 2020 in workshops in East Lombok, Indonesia, and 2 weeks within December 2020 in Kaffrine, Senegal.

The PEX:FGDs were kept open-ended so that participants could dictate the direction of the discussion, which allowed for topics that may not have been pre-considered by the facilitators to arise. Sessions were facilitated by local indigenous researchers, guided in process by researchers more experienced in the approach, and were carried out in the local languages, Bahasa in East Lombok, Indonesia and French or Wolof in Kaffrine, Senegal.

### Development of localised *WVIS* materials

Important to the *WVIS* approach is the development of localised materials (Table 1). The main trigger list has been found applicable in globalised places where English is the first language, but otherwise the trigger lists are locally generated in the local language, incorporating local vocabulary and ways of thinking. To generate these, 5–8 specific interviews are taken with local community members, by indigenous university researchers, eliciting local phrases and ways of thinking. This is a necessary step because shared tacit values cannot be easily accessed without using local language. Examples of localised Trigger Statements produced this way are given below: (they all start with: “It is important to me/us that…”):

- …there is solidarity and mutual aid between the people
- …I can still be in communication with my children, even if far away
- …husbands are responsible for the care of their wives and family
- …the town council fulfils its responsibility to meet our needs
- …people are not afraid of hard, and even manual work

### Study participants

The group participants targeted for recruitment, were selected by local country Hub co-researchers to meet two sets of requirements. For suitability for the *WVIS* approach they should be between 3 and 12 in number; belong to naturally existing groups that have some history of shared experiences; are over 18 years old; do not include members holding significantly more power than others; and speak the same native language. For suitability in the PEX:FGD to offer life practices with relevance to the research topic of stunting, the groups were chosen to represent stakeholders with connections to the food or learning environment of children (which the Action Against Stunting Hub refer to as the Whole Child approach) [33]. The university researchers specialising in shared values from the UK, and Senegal and Indonesia respectively, discussed together which stakeholder groups might be appropriate to recruit. The local researchers made the final decisions. Each group was taken through both a *WVIS* workshop and the immediately-subsequent PEX:FGD.

### Data collection and analysis

**Fig. 2:**
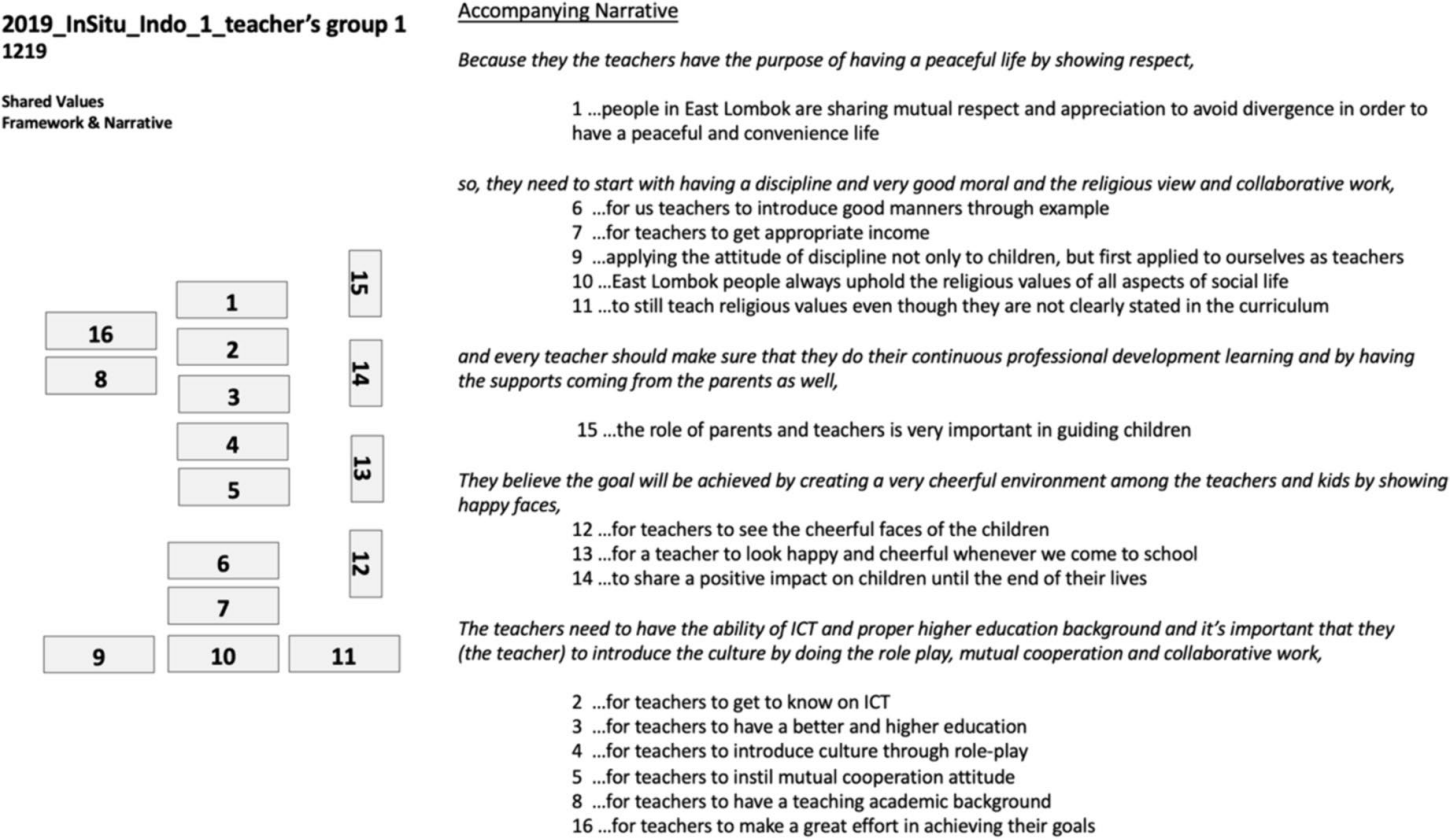
An illustrative example of one digitized Shared Values Framework and accompanying Narrative from a teacher’s group in East Lombok, Indonesia. The “…” refers to each statement being preceded by “It is important to us that…”

Standard data output from the WeValue session includes i) the jointly-negotiated bespoke Statements of shared values, linked together in their unique Framework, and ii) an oral recording of a descriptive Narrative of it, given by the group. These were digitized to produce a single presentation for each group as in Fig. 2. It represents the synthesised culmination of the crystallisation process: a portrait of what was ‘important’ to each stakeholder group. Separately, statements from the group about the authenticity/ownership of the statements are collected.

When these Frameworks of ‘Statements of Shared Values’ are viewed *across all the groups* from one locality (Locality Shared Values Statements), they provide portraits of ‘what is important’ to people living there, often in intimate detail and language. They can be used to communicate to ‘outsiders’ what the general cultural shared values are. In this work the researchers thematically coded them using Charmaz constructionist grounded theory coding [35] to find broad Major Cultural Themes within each separate locality.

The second area of data collection was in the post-*WVIS* event: the PEX:FGD for each group. A translator/interpreter provided a running commentary during these discussions, which was audio recorded and then transcribed. The specific topics raised for each group to discuss varied depending on their local expertise. This required completely separate workstreams of coding of the dataset with respect to each topic. This was carried out independently by two researchers: one from UK (using NVivo software (Release 1.3.1)) and one from the local country, who resolved any small differences. All the transcripts were then collated and inductively, interpretively analysed to draw out insights that should be relayed back to the Action Against Stunting Hub teams as contextual material.

The extracts of discussion which were identified as relevant within a particular Hub theme (e.g. hygiene) were then meta-ethnographically synthesised [36] into ‘Hub Theme Statements’ on each topic, which became the core data for later communication and interrogation by other researchers within the Action Against Stunting Hub. These statements are interpretations of participants’ intended meanings, and links from each of them to data quotes were maintained, enabling future interpretations to refer to them for consistency checks between received and intended meaning.

In this investigation, those Hub Theme Statements (derived from PEX:FGD transcripts) were then deductively coded with respect to any topics with potential implications of the egg intervention. Literature regarding barriers and facilitators to nutrition interventions indicated the following topics could be relevant: attitudes to accepting help; community interactions; cooking and eating habits; traditional beliefs about malnutrition; sharing; social hierarchies [12, 37, 38] to which we added anything related to pregnancy or eggs. This analysis produced our Egg Intervention Themes from the data.

**Fig. 3:**
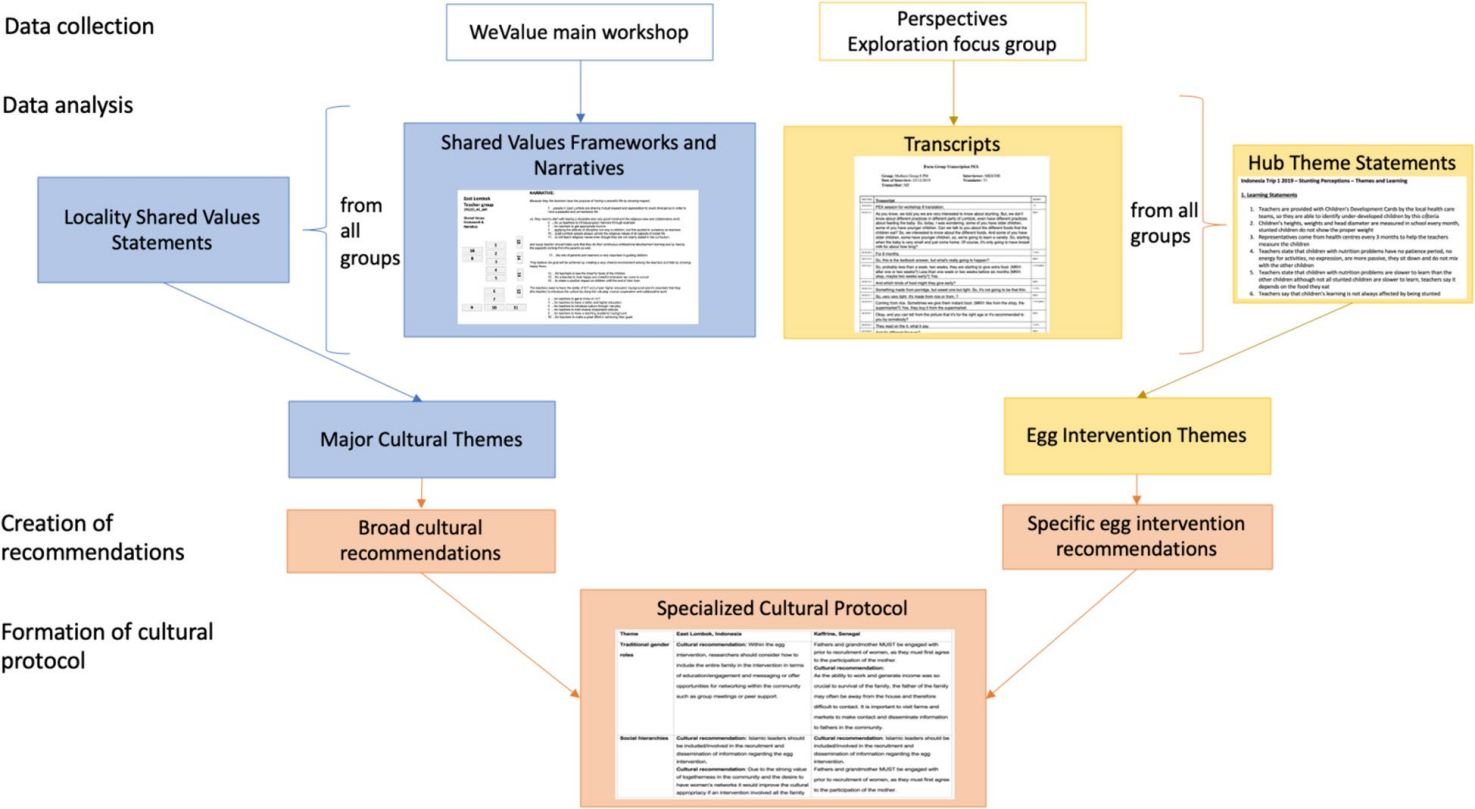
Schematic representation of the method of production of the culturally-informed protocol for each locality

The Major Cultural Themes and Egg Intervention Themes were then used to create a set of culture-based recommendations and intervention specific recommendations respectively for each locality. These recommendations were then combined to form specialized culturally-informed protocols for the egg intervention in each locality: East Lombok, Indonesia and Kaffrine, Senegal. The process is displayed schematically in Fig. 3.

## Results

The preparation of the localised *WVIS* materials at each site took 6 hours of interview field work, and 40 person hours for analysis. The 10 workshops and data summaries were concluded within 10 workdays by two people (80 person hours). The analysis of the PEX:FGD data took a further 80 person hours. Thus, the total research time was approximately 200 person hours.

The stakeholder group types are summarised in Table 2. The data is presented in three parts. Firstly, the Major Cultural Themes found in East Lombok, Indonesia and in Kaffrine, Senegal are described – the ones most heavily emphasised by participants. Then, the Egg Intervention Themes and finally, the combined set of Recommendations to comprise a culturally-informed protocol for intervention design for each location. Quotations are labelled INDO or SEN for East Lombok, Indonesia and Kaffrine, Senegal, respectively.

**Table 2:** The number of participants and stakeholder group types for both Indonesia and Senegal

Type of Group	East Lombok, Indonesia		Kaffrine, Senegal	
	No. of Groups	Total No. of Participants	No. of Groups	Total No. of Participants
**Total**	**9**	**39**	**11**	**44**
Mothers	6	25	3	12
Teachers	2	10	1	4
Fathers	1	4	1	4
Grandmothers	0	0	1	4
Market traders	0	0	1	4
Administrators	0	0	1	4
Community Health Workers	0	0	2	8
Farmers	0	0	1	4

**Table 3:** Integrated Egg Intervention recommendations synthesized from the PEXs and the Frameworks and Narratives for East Lombok (Indonesia) and Kaffrine (Senegal). Where elements of the recommendation are derived from Major Cultural Themes, this has been noted as Cultural recommendation, highlighted in bold

Theme	East Lombok, Indonesia	Kaffrine, Senegal
**Attitudes to accepting outside help**	**Culture-based recommendation:** Participants and their families should be provided with plentiful culturally relevant information resources for them to learn about stunting and healthy nutrition, in order to encourage menu changes.	Fathers should be engaged with early in order to increase the likelihood that they will allow for their wives to participate. Men of influence in the community such as Islamic leaders could be asked to help with this level of engagement to increase trust and likelihood of agreement to participate by men. The Government could raise its profile as a supporter of nutrition or be invited/contacted to collaborate in messaging dissemination. The Egg Intervention might need to have eggs consumed in presence of health worker (and thus pre-prepared) to ensure it is not stored for later or other use.
**Traditional gender roles**	**Culture-based recommendation:** Within the egg intervention, researchers should consider how to include the entire family in the intervention in terms of education/engagement and messaging or offer opportunities for networking within the community such as group meetings or peer support.	Fathers and grandmother MUST be engaged with prior to recruitment of women, as they must first agree to the participation of the mother. **Culture-based recommendation:** As the ability to work and generate income was so crucial to survival of the family, the father of the family may often be away from the house and therefore difficult to contact. It is important to visit farms and markets to make contact and disseminate information to fathers in the community.
**Social hierarchies**	**Culture-based recommendation:** Islamic leaders should be included/involved in the recruitment and dissemination of information regarding the egg intervention. **Culture-based recommendation:** Due to the strong value of togetherness in the community and the desire to have women’s networks it would improve the cultural appropriacy if an intervention involved all the family or had an element of group interaction for example breastfeeding support groups for women.	**Culture-based recommendation:** Islamic leaders should be included/involved in the recruitment and dissemination of information regarding the egg intervention. Fathers and grandmother MUST be engaged with prior to recruitment of women, as they must first agree to the participation of the mother.
**Food sharing**	Specific messaging about how the egg should be prepared and potential problems with sharing should be disseminated in order to encourage the consumption of the egg by the mother. Egg consumption may need to be supervised by a community health worker to ensure it is consumed by the participant. Additional support could be provided such as information leaflets for additional family members.	Specific messaging about how the egg should be prepared and the problems with sharing should be disseminated in order to encourage the consumption of the egg by the mother. Egg consumption may need to be supervised by a community health worker to ensure it is consumed by the mother. Additional support could be provided such as information leaflets for additional family members.
**Traditional beliefs about malnutrition**	**Culture-based recommendation:** Interventions should provide plentiful culturally appropriate messaging about undernutrition and stunting with nutritional advice taking into account cultural diets and limitations of affordability of certain foods. Interventions to improve infant and child feeding practices should offer support to breastfeeding mothers, perhaps through group education sessions with lactation consultants.	The intervention should include an element of education so that families with affected children can learn the determinants/risk factors for stunting and the impacts of traditional beliefs can be mitigated. Engagement should include both grandmothers and mothers.
**Pregnancy beliefs**	**Culture-based recommendation:** Notice should be taken of unmarried pregnant women within the community who may face stigma and be less likely to approach providers of an intervention. Encouragement of participation could be carried out through community outreach.	Notice should be taken young pregnant women, or women with young children within the community (close pregnancies) who may face stigma and be less likely to approach providers of an intervention. Encouragement of participation should be carried out through appropriate community outreach. Messaging and communications within the information provided should address misperceptions around healthy foods for pregnant women related to traditional beliefs. Engagement should include both grandmothers and mothers.
**Specific views on eggs**	The intervention should take into account the perception of eggs as a healthy food, and that they are already commonly used. It must be emphasised to mothers that the supplemented egg is additional to their existing diet.	The intervention should include an element of educational messaging to dispel misperceptions around the consequences of eating eggs while pregnant and inform participants of the benefits of consuming eggs. Since this belief is traditional, this education should include older family members such as those grandmothers who have power over the decision-making in the family.
**Other – food practices**	**Culture-based recommendation:** Due to the communal meal being cooked and then eaten throughout the day – note must be taken of the likelihood of food sharing. Specific messaging about how the egg should be prepared and the problems with sharing should be disseminated. Egg consumption may need to be supervised by a community health worker to ensure it is consumed by the participant. This should be handled sensitively with guidance from local researchers.	**Culture-based recommendation:** Due to the communal nature of mealtimes, notice must be taken of sharing and that the patriarch may take food first. Messaging should encourage mothers to eat the supplemented egg themselves and emphasise that this should not be shared. Egg consumption may need to be supervised by a community health worker to ensure it is consumed by the participant. This should be handled sensitively with guidance from local researchers.
**Other – Access to health services**		Mitigate access challenges with home visits. **Culture-based recommendation:** Fathers should be engaged with early, in order to increase the likelihood that they will allow for their wives to participate. Men of influence in the community such as Islamic leaders could be asked to help with this level of engagement to increase trust and likelihood of agreement to participate by men.

### Major cultural themes from frameworks and narratives

These were derived from the Locality Shared Values Statements produced in the *WVIS*.

#### East Lombok, Indonesia

##### Religious values

Islamic values were crucially important for participants from East Lombok, Indonesia and to their way of life. Through living by the Quran, participating in Islamic community practices, and teaching Islamic values to their children, participants felt they develop their spirituality and guarantee a better afterlife for themselves and their children. Participants stated the Quran tells them to breastfeed their children for 2 years, so they do. Despite no explicit religious official curriculum in Kindergarten, the teachers stated that it was important to incorporate religious teaching.

*“East Lombok people always uphold the religious values of all aspects of social life.”*

*“It is important for me to still teach religious values even though they are not clearly stated in the curriculum.”* – Workshop 1 INDO (teachers).

*“In Quran for instance, we are told to breastfeed our kids for 2 years. We can even learn about that*” – Workshop 3 INDO (mothers).

Related to this was the importance of teaching manners to children and preventing them from saying harsh words. Teachers stated that it was important to create a happy environment for the children and to ensure that they are polite and well-behaved. Similarly, mothers emphasised the need to teach their children good religious values to ensure they will be polite and helpful to their elders.

*“Children don’t speak harsh words.”*

*“My children can help me like what I did to my parents”.*

– Workshop 8 INDO (mothers).

##### Togetherness within families and the community

The Locality Shared Values Frameworks stressed the importance of togetherness, both within family and community. Comments mentioned it being important that people rely heavily on their family and come together in times of need to support each other and provide motivation. This was also important more broadly, in that people in society should support each other, and that children grow up to contribute to society. This was also reflected in comments around roles within the family. Despite women being primary care givers, and men working to finance the family, participants stated that they follow a process of consultation to make decisions, and when facing hardships.

*“that we have the sense of kinship throughout our society”.*

*“We have togetherness as mothers”.*

– Workshop 8 INDO (mothers).

*“For the family side, whatever happens we need to be able to be united as a whole family. We need to have the [sense of] forgiveness for the sake of the children” –* Workshop 2 INDO (mothers).

##### Attitudes about extra-marital pregnancy

In East Lombok, Indonesia, it was essential to both mothers and fathers that pregnancy happened within a marriage, this was to ensure that the honour of the family was upheld and that the lineage of the child was clear. The potential danger to health that early pregnancies can cause was also acknowledged.

*“If they don’t listen to parents’ advice, there will be the possibility of pre-marital pregnancy happening, which will affect the family [so much].*

*The affect is going to be ruining the good name, honour and family dignity. When the children [are] born outside [of] marriage, she or he will have many difficulties like getting a birth certificate [and] having a hard time when registering to school or family” - Workshop 4 INDO (mothers).*

“*To make sure that our children avoid getting married at a very young age and moreover [avoid] having free sex so that they will not get pregnant before the marriage”* - Workshop 9 INDO (fathers).

#### Kaffrine, Senegal

The Major Cultural Themes which emerged from the Kaffrine data are described below. As these are grounded themes, they are different than those seen in East Lombok, Indonesia.

##### Access to healthcare

A recurring theme amongst the groups in Kaffrine were aspirations of affordable and easy-to-access healthcare. Community health workers stated the importance of encouraging women to give birth in hospitals and spoke of the importance of preventing early pregnancy which result from early marriages. Giving birth in hospitals was also a concern for Public Office Administrators who highlighted that this leads to subsequent issues with registering children for school. Mothers and fathers stated the importance of being able to afford health insurance and access healthcare so that they could take care of themselves.

*“That the women give birth in the hospital” –* Workshop 11 SEN (CHWS).

*“To have affordable health insurance*” – Workshop 10 SEN (mothers).

*“To have access to health care*” – Workshop 3 SEN (fathers).

*“It is important that women give birth in the hospital in order to be able to have a certificate that allows us to establish the civil status” –* Workshop 9 SEN (administrators).

Additionally, Community health workers spoke of their aspiration to have enough supplements to provide to their community so as to avoid frustration at the lack of supply, and mothers spoke of their desire to be provided with supplements.

*“To have dietary supplements in large quantities to give them to all those who need them, so as not to create frustration” –* Workshop 11 SEN (CHWS).

Another aspect of access to healthcare, was mistrust between fathers and community health workers. Community health workers explained that sometimes men can blame them when things go wrong in a pregnancy or consider their ideas to be too progressive. Thus, to these community health workers the quality of endurance was very important.

*“Endurance (Sometimes men can accuse us of influencing their wives when they have difficulties in conceiving)”* – Workshop 5 SEN (CHWs).

##### Employment

Another recurring theme was the importance of having secure employment and a means to support themselves; that there were also jobs available for young people, and that women had opportunities to make money to help support the family. This included preventing early marriages so girls could stay in school. Having jobs was stated as essential for survival and important to enable being useful to the community and society.

*“To have more means of survival (subsistence) to be able to feed our families”.*

*“To have a regular and permanent job”.*

*“We assure a good training and education for our children so that they will become useful to us and the community”.*

“*Our women should have access to activities that will support us and lessen our burden”* – Workshop 3 SEN (fathers).

##### Religious values

It was considered very important to have a religious education and respect for religious elders. Moreover, living by, and teaching, religious values such as being hard working, humble and offering mutual aid to others, was significant for people in Kaffrine.

*“Have an education in the Islamic Culture (Education that aligns with the culture of Islam)”.*

*“Respect toward religious leaders”* – Workshop 3 SEN (fathers).

“*To organize religious discussions to develop our knowledge about Islam*” - Workshop 10 SEN (mothers).

“*Have belief and be prayerful and give good counselling to people*” - Workshop 4 SEN (grandmothers).

### Egg intervention themes from each country from perspectives EXplorations focus group discussion data

Below are results of analyses of comments made during the PEX:FGDs in East Lombok, Indonesia and Kaffrine, Senegal. The following codes were used deductively: attitudes to accepting outside help, traditional gender roles, food sharing, traditional beliefs, social hierarchies and understanding of stunting and Other. These topics were spoken about during open discussion and were not the subject of direct questions. For example, topics relating to traditional gender roles came up in East Lombok, during conversations around the daily routine. Thus, in order to more accurately reflect the intended meaning of the participants, these were labelled food practices, under the “Other” theme. If any of the themes were not present in the discussion, they are not shown below.

#### East Lombok, Indonesia

##### Attitudes to accepting outside help

Few mentions were made that focussed on participants attitudes to accepting outside help, but participants were sure that they would not make changes to their menus based on the advice of outside experts. Additionally, teachers mentioned that they are used to accepting help from local organisations that could to help them to identify under-developed children.

“*We don’t believe that [the outsiders are] going to change our eating habits or our various menus*” – Workshop 3 INDO (Mothers).

##### Traditional gender roles

In East Lombok, mothers spoke about how their husbands go to work and then provide them with daily money to buy the food for the day. However, this was discussed in relation to why food is bought daily and is thus discussed below in the topics Other – Food practices.

##### Food sharing

In East Lombok, Indonesia, in times when they have extra food, they share it with neighbours, in the hope that when they face times of hardship, their neighbours will share with them. Within the household, they mentioned sharing food from their plate with infants and encouraging children to share. Some mothers mentioned the importance of weekly meetings with other mothers to share food and sharing food during celebrations.

“*Sometimes we share our food with our family. So, when we cook extra food, we will probably send over the food to our neighbour, to our families. So, sometimes, with the hope that when we don’t have anything to eat, our neighbour will pay for it and will [share with] us.”* – Workshop 3 INDO (Mothers).

*“Even they serve food for the kids who come along to the house. So, they teach the kids to share with their friends. They provide some food. So, whenever they play [at their] house, they will [eat] the same.” –* Workshop 2 INDO (Mothers).

##### Understanding of stunting

The teachers in East Lombok were aware of child stunting through Children’s Development Cards provided by local healthcare organizations. They stated that they recognise children with nutrition problems as having no patience period, no expression, no energy for activities and less desire to socialise and play with other children. The teachers said that stunted children do not develop the same as other children and are not as independent as children who are the proper height and weight for their development. They also stated that they recognise stunted children by their posture, pale faces and bloated stomachs. They explained how they usually use the same teaching methods for stunting children, but will sometimes allow them to do some activities, like singing, later, once the other children are leaving.

“*They have no patience period, don’t have any energy to do any of the activities. No expression, only sitting down and not mingling around with the kids. They are different way to learn. They are much slower than the other kids*.” – Workshop 1 INDO (teachers).

“*When they are passive in singing, they will do it later when everyone else is leaving, they just do it [by] themselves*” – Workshop 1 INDO (teachers).

##### Specific views on eggs

In East Lombok, Indonesia, there were no superstitions or traditional beliefs around the consumption of eggs. When asked specifically on their views of eggs, and if they would like to be provided with eggs, women in East Lombok said that they would be happy to accept eggs. They also mentioned that eggs were a food they commonly eat, feed to children and use for convenience. Eggs were considered healthy and were common in their house.

“*We choose eggs instead. If we don’t have time, we just probably do some omelettes or sunny side up. So, it happens, actually when we get up late, we don’t have much time to be able to escort our kids to the school, then we fry the eggs or cook the instant noodles. And it happens to all mothers. So, if my kids are being cranky, that’s what happens, I’m not going to cook proper meals so, probably just eggs and instant noodles.”* – Workshop 3 INDO (Mothers).

##### Other important topics – food practices

Some detailed themes about food practices were heard in East Lombok, Indonesia. The women were responsible for buying and preparing the food, which they purchased daily mainly due to the cost (their husbands were paid daily and so provided them with a daily allowance) and lack of storage facilities. They also bought from mobile vendors who came to the street, because they could buy very small amounts and get occasional credit. The mother decided the menu for the family and cooked once per day in the morning: the family then took from this dish throughout the day. Mothers always washed their fruits and vegetables and tried to include protein in their meals when funds allowed: either meat, eggs, tofu or tempeh.

“*One meal a day. They* [the mothers] *cook one time and they* [the children] *can eat it all day long. Yes, they can take it all day long. They find that they like [to take the food], because they tend to feel hungry.”* – Workshop 6 INDO (Mothers).

“*They shop every day because they don’t have any storage in their house and the other factor is because the husband has a daily wage. They don’t have monthly wage. In the morning, the husband gives the ladies the money and the ladies go to the shop for the food.*” – Workshop 4 INDO (Mothers).

#### Kaffrine, Senegal

In Kaffrine, the following themes emerged relating to an egg intervention: they were different in content and emphasis to Lombok and contained uniquely local cultural emphases.

##### Attitudes to accepting outside help

Mothers were welcoming of eggs as a supplement to improve their health during pregnancy and acknowledged the importance of good nutrition during pregnancy. However, they also mentioned that their husbands can sometimes be resistant to accepting outside help and provided an example of a vaccination programme in which fathers were hesitant to participate. However, participants stated that the Government should be the source of assistance to them (but currently was not perceived to be so).

*“But if these eggs are brought by external bodies, we will hesitate to take it. For example, concerning vaccination some fathers hesitate to vaccinate their children even if they are locals who are doing it. So, educating the fathers to accept this is really a challenge” –* Workshop 11 SEN (CHWs).

##### Traditional gender roles

Some traditional gender roles were found to be strong. The participants emphasised that men are considered the head of the household, as expected in Islam, with the mother as primary caregiver for children. This is reflected in the comments from participants regarding the importance of Islam and living their religious values. The men thus made the family decisions and would need to be informed and agree to any family participation in any intervention – regardless of the education level of the mother. The paternal grandmother also played a very important role in the family and may also make decisions for the family in the place of the father. Community Health Workers emphasised that educating paternal grandmothers was essential to improve access to healthcare for women.

*“There are people who are not flexible with their wives and need to be informed. Sometimes the mother-in-law can decide the place of the husband. But still, the husband’s [permission] is still necessary.” –* Workshop 1 SEN (CHWs).

*“[We recommend] communication with mothers-in-law and the community. Raise awareness through information, emphasizing the well-being of women and children.” –* Workshop 1 SEN (CHWs).

*“The [grand]mothers take care of the children so that the daughters in-law will take care of them in return So it’s very bad for a daughter in law not to take care of her mother in-law. Society does not like people who distance themselves from children.” –* Workshop 4 SEN *(grandmothers).*

##### Social hierarchies

In addition to hierarchies relating to gender/position in the family such as grandmothers have decision making power, there was some mention of social hierarchies in Kaffrine, Senegal. For example, during times of food stress it was said that political groups distribute food and elected officials who choose the neighbourhoods in which the food will be distributed. Neighbourhood leaders then decide to whom the food is distributed, meaning there is a feeling that some people are being left out.

“*It’s political groups that come to distribute food or for political purposes…organizations that often come to distribute food aid, but in general it is always subject to a selection on the part of elected officials, in particular the neighbourhood leaders, who select the people they like and who leave the others*” – Workshop 11 SEN (CHWs).

##### Food sharing

Participants explained that during mealtimes, the family will share food from one large plate from which the father will eat first as a sign of respect and courtesy. Sometimes, children would also eat in their neighbour’s house to encourage them to eat.

“*Yes, it happens that we use that strategy so that children can eat. Note that children like to imitate so that’s why we [send them to the neighbour’s house]”* – Workshop 11 SEN (CHWs)”.

##### Traditional beliefs about malnutrition

In Kaffrine, Senegal, some participants spoke of traditional beliefs relating to malnutrition, which are believed by fewer people these days. For example, uncovered food might attract bad spirits, and any person who eats it will become ill. There were a number of food taboos spoken of which were thought to have negative consequences for the baby, for example watermelon and grilled meat which were though to lead to birth complications and bleeding. Furthermore, cold water was thought to negatively impact the baby. Groups spoke of a tradition known as “bathie” in which traditional healers wash stunted children with smoke.

“*There are traditional practices called (Bathie) which are practiced by traditional healers. Parents are flexible about the practice of Bathie*” – Workshop 1 SEN (CHWs).

##### Understanding of stunting

Causes of malnutrition and stunting were thought to be a lack of a balanced diet, lack of vitamin A, disease, intestinal worms, poor hygiene, socio-cultural issues such as non-compliance with food taboos, non-compliance with exclusive breastfeeding and close pregnancies. Malnutrition was also thought by some to be hereditary. Numerous signs of malnutrition were well known amongst the groups in Kaffrine. For example, signs of malnutrition were thought to be a big bloated belly, diarrhoea, oedema of the feet, anaemia, small limbs and hair loss as well as other symptoms such as red hair and a pale complexion. Despite this, malnutrition was thought to be hard to identify in Kaffrine as not all children will visit health centres, but mothers do try to take their babies heights and weights monthly. The groups were aware of the effect of poverty on the likelihood of stunting as impoverished parents cannot afford food. Furthermore, the groups mentioned that there is some stigma towards stunted children, and they can face mockery from other children although most local people feel pity and compassion towards them. Malnourished children are referred to as Khiibon or Lonpogne in the local language of Wolof.

“*It is poverty that is at the root of malnutrition, because parents do not have enough money [and] will have difficulty feeding their families well, so it is the situation of poverty that is the first explanatory factor of malnutrition here in Kaffrine” –* Workshop 9 SEN (administrators).

*“It can happen that some children are the victim of jokes for example of mockery from children of their same age, but not from adults and older*” *–* Workshop 9 SEN (administrators).

##### Pregnancy beliefs

In Kaffrine, Senegal, there were concerns around close pregnancies, and pregnancies in women who were too young, and for home births. Within the communities there was a stigma around close pregnancies, which prevented them from attending antenatal appointments. Similarly, there were superstitions around revealing early pregnancies, which again delayed attendance at health centres.

Groups acknowledged the role of good nutrition, and mentioned some forbidden foods such as salty foods, watermelon and grilled meat (which sometimes related back to a traditional belief that negative impacts would be felt in the pregnancy such as birth complications and bleeding). Similarly, drinking cold water was thought to negatively affect the baby. Beneficial foods mentioned included vegetables and meat, during pregnancy.

“*Often when a woman has close pregnancies, she can be ashamed, and this particularly delays the time of consultation” –* Workshop 5 SEN (CHWs).

*“Yes, there are things that are prohibited for pregnant women like salty foods” –* Workshop 11 SEN (CHWs).

##### Specific views on eggs

In Kaffrine, Senegal, some participants spoke of a traditional belief that if a pregnant woman consumes eggs then her baby might be overweight, or have problems learning how to talk. Despite this, mothers in Kaffrine said that they would be happy to accept eggs as a supplement, although if supplements are provided that require preparation (such as powdered supplements), they would be less likely to accept them.

*“These restrictions are traditional, and more women no longer believe that eggs will cause a problem to the child. But if these eggs are brought by external bodies, we will hesitate to take it.” –* Workshop 11 SEN (CHWs).

*“They don’t eat eggs before the child starts speaking (the child only eats eggs when he starts talking). This is because it’s very heavy and can cause bloating and may also lead to intestinal problems.”* – Workshop 4 SEN *(grandmothers).*

##### Other important topics – access to health services

For the participants in Kaffrine, Senegal, accessing health services was problematic, particularly for pre- and post-natal appointments, which faced frequent delays. Some women had access due to poor roads and chose to give birth at home. Access issues were further compounded by poverty and social factors, as procedures in hospitals can be costly, and women with close pregnancies (soon after an earlier one) can feel shame from society and hide their pregnancy.

*“Women really have problems of lack of finances. There are social services in the hospital; but those services rarely attend to women without finances. Even when a child dies at birth they will require money to do the necessary procedure*” – Workshop 11 SEN (CHWs).

### Creation of the culturally-informed protocols

Recommendations that comprise a culturally-informed protocol for intervention design in each locality are given in Table 3.

## Discussion

The Major Cultural Themes, and specific Egg Intervention Themes drawn out from only 9–11 carefully planned group sessions in each country provided a rich set of recommendations towards a culturally-informed protocol for the localised design of a proposed Egg Intervention for both East Lombok, Indonesia and Kaffrine, Senegal. A culturally-informed protocol designed in this way comprises cultural insights which are worthy of consideration in local intervention design and should guide future stages of engagement and provide a platform from which good rapport and trust can be built between researchers and the community [16]. For example, in Kaffrine, Senegal, the early involvement of husbands and grandmothers is crucial, which reflects values around shared decision making within families that are noted to be more prevalent in LMICs, in contrast to individualistic values in HICs [16, 39]. Similarly, due to strong religious values in both East Lombok, Indonesia and Kaffrine, Senegal, partnerships with Islamic leaders is likely to improve engagement. Past studies show the crucial role that religious leaders can play in determining social acceptability of interventions, particularly around taboo topics such as birth spacing [40].

The *WVIS* plus PEX:FGD method demonstrated here produced both broad cultural themes from shared values, which were in a concise and easy-to-understand format which could be readily communicated with the wider Action Against Stunting Hub, as well as life practices relevant to stunting in Kaffrine, Senegal and in East Lombok, Indonesia. Discussions of shared values during the *WVIS* main workshop provided useful cultural background within each community. PEX:FGD discussion uncovered numerous cultural factors within local life practices that could influence on the Egg Intervention engagement and acceptability. Combining themes from the *WVIS* workshop and PEX:FGDs allowed for specific recommendations to be made towards a culturally-informed protocol for the design of an Egg Intervention that included both broad cultural themes and specific Intervention insights (Table 3). For example, in Kaffrine, Senegal, to know that the husband’s authoritative family decision-making for health care (specific) is rooted in Islamic foundations (wider cultural) points to an Intervention Recommendation within the protocol, involving consultations with Islamic Leaders to lead community awareness targeting fathers. Similarly, in East Lombok, Indonesia the (specific) behaviour of breastfeeding for 2 years was underpinned by (wider cultural) shared values of living in Islam. This understanding of local values could prevent the imposition of culturally misaligned values, which Bernal and Adames (2017) caution against [17].

There are a number of interesting overlaps between values seen in the *WVIS* Frameworks and Narratives and the categories of Schwartz (1992) and The World Values Survey (2023) [41, 42]. For example, in both Kaffrine, Senegal and East Lombok, Indonesia, strong religious values were found, and the groups spoke of the importance of practicing their religion with daily habits. This would align with traditional and conservation values [41, 43]. Furthermore, in Kaffrine, Senegal participants often mentioned the importance of mutual aid within the community, and similar values of togetherness and respect in the community were found in East Lombok, Indonesia. These would seem to align with traditional, survival and conservation values [41, 43]. However, the values mentioned by the groups in the *WVIS* workshops are far more specific, and it is possible that through asking what is most worthwhile, valuable and meaningful about their context, the participants are able to prioritise which aspects of their values are most salient to their daily lives. Grounded shared values such as these are generally neglected in Global Health Research, and values predominant in the Global North are often assumed to be universal [14]. Thus, by excluding the use of a predefined external framework, we minimized the risk of imposing our own ideas of values in the community, and increased the relevance, significance and local validity of the elicited information [28].

Participatory methods of engagement are an essential step in conducting Global Health Research but there is currently a paucity of specific guidance for implementing participatory methods in vulnerable communities [16, 44]. In addition, there is acknowledgement in the literature that it is necessary to come into communities in LMICs without assumptions about their held values, and to use bottom-up participatory approaches to better understand local values [14, 16]. The *WVIS* plus PEX:FGD methodology highlighted here exemplifies a method that is replicable in multiple country contexts [28, 32] and can be used to crystallize local In Situ Shared Values which can be easily communicated to external researchers. Coupled with the specialised FGD (PEX:FGD), values-based perceptions of specific topics (in this case stunting) can be elicited leading to the creation of specific Culture-based recommendations. This therefore takes steps to answer the call by Memon and colleagues (2021) for the creation of cultural protocols ahead of conducting research in order to foster ethical research relationships [16]. We believe that the potential usefulness of the *WVIS* approach to guide engagement and inform intervention design is effectively demonstrated in this study and *WVIS* offers a method of making explicit local values in a novel and valuable way.

However, we acknowledge that our approach has several limitations. It has relied heavily on the local university researchers to debate and decide which participant stakeholder groups should be chosen, and although they did this in the context of the Whole Child approach, it would have been advantageous to have involved cultural researchers with a deeper understanding of cultural structures, to ensure sufficient opportunities for key cultural elements to emerge. This would have in particular strengthened the intervention design derived from the PEX:FGD data. For example, we retrospectively realised that our study could have been improved if grandmothers had been engaged in East Lombok. Understanding this limitation leads to suggestion for further work: to specifically investigate the overlap of this approach with disciplinary studies of culture, where social interactions and structures are taken into account via formal frameworks.

There are more minor limitations to note. For example, the *WVIS* approach can only be led by a trained and experienced facilitator: not all researchers can do this. A training programme is currently under development that could be made more widely available through online videos and a Handbook. Secondly, although the groups recruited do not need to be representative of the local population, the number recruited should be increased until theoretical saturation is achieved of the themes which emerge, which was not carried out in this study as we focussed on demonstrating the feasibility of the tool. Thirdly, there is a limit to the number of topics that can be explored in the PEX:FGDs within the timeframe of one focus group (depending on the stamina of the participants), and so if a wider range of topics need formative research, then more workshops are needed. Lastly, this work took place in a large, highly collaborative project involving expert researchers from local countries as well as international experts in *WVIS*: other teams may not have these resources. However, local researchers who train in *WVIS* could lead on their own (and in this Hub project such training was available).

## Conclusion

The need for better understanding, acknowledgement and integration of local culture and shared values is increasing as the field of Global Health Research develops. This study demonstrates that the *WVIS* plus PEX:FGD shared values approach provides an efficient approach to contextualise and localise interventions, through eliciting and making communicable shared values and local life practices which can be used towards the formation of a culturally-informed protocols. Were this method to be used for intervention design in future, it is possible that more focus should be given to existing social structures and support systems and a greater variety of stakeholders should be engaged. This study thus contributes to the literature on methods to culturally adapt interventions. This could have significant implications for improving the uptake of nutrition interventions to reduce malnutrition through improved social acceptability, which could help progression towards the goal of Zero Hunger set within the SDGs. The transferability and generalisability of the *WVIS* plus PEX:FGD approach should now be investigated further in more diverse cultures and for providing formative research information for a wider range of research themes. Future studies could also focus on establishing its scaling and pragmatic usefulness as a route to conceptualising mechanisms of social acceptability, for example a mechanism may be that in communities with strong traditional religious values, social hierarchies involving religious leaders and fathers exist and their buy-in to the intervention is crucial to its social acceptability. Studies could also focus on the comparison or combination of *WVIS* plus PEX:FGD with other qualitative methods used for intervention design and implementation.

## Data Availability

The datasets used and/or analysed during the current study are available from the corresponding author on reasonable request m.k.harder@brighton.ac.uk, Orcid number 0000–0002–1811-4597. These include deidentified Frameworks of Shared Values and Accompanying Narrative from each Group; deidentified Hub Insight Statements of relevant themes.
